# A Latent Factor Analysis of Working Memory Measures Using Large-Scale Data

**DOI:** 10.3389/fpsyg.2017.01062

**Published:** 2017-06-28

**Authors:** Otto Waris, Anna Soveri, Miikka Ahti, Russell C. Hoffing, Daniel Ventus, Susanne M. Jaeggi, Aaron R. Seitz, Matti Laine

**Affiliations:** ^1^Department of Psychology, Åbo Akademi UniversityTurku, Finland; ^2^Turku Brain and Mind Center, University of TurkuTurku, Finland; ^3^Department of Psychology, University of TurkuTurku, Finland; ^4^Department of Psychology, University of California, Riverside, RiversideCA, United States; ^5^School of Education, University of California, Irvine, IrvineCA, United States

**Keywords:** working memory, latent variable, confirmatory factor analysis, exploratory factor analysis, simple span, complex span, running memory task, *n*-back

## Abstract

Working memory (WM) is a key cognitive system that is strongly related to other cognitive domains and relevant for everyday life. However, the structure of WM is yet to be determined. A number of WM models have been put forth especially by factor analytical studies. In broad terms, these models vary by their emphasis on WM contents (e.g., visuospatial, verbal) vs. WM processes (e.g., maintenance, updating) as critical, dissociable elements. Here we conducted confirmatory and exploratory factor analyses on a broad set of WM tasks, half of them numerical-verbal and half of them visuospatial, representing four commonly used task paradigms: simple span, complex span, running memory, and *n*-back. The tasks were selected to allow the detection of both content-based (visuospatial, numerical-verbal) and process-based (maintenance, updating) divisions. The data were collected online which allowed the recruitment of a large and demographically diverse sample of adults (*n* = 711). Both factor analytical methods pointed to a clear division according to task content for all paradigms except *n*-back, while there was no indication for a process-based division. Besides the content-based division, confirmatory factor analyses supported a model that also included a general WM factor. The *n*-back tasks had the highest loadings on the general factor, suggesting that this factor reflected high-level cognitive resources such as executive functioning and fluid intelligence that are engaged with all WM tasks, and possibly even more so with the *n*-back. Together with earlier findings that indicate high variability of process-based WM divisions, we conclude that the most robust division of WM is along its contents (visuospatial vs. numerical-verbal), rather than along its hypothetical subprocesses.

## Introduction

Working memory (WM) is a capacity-limited short-term memory system that is engaged in the processing of currently active information (e.g., Conway et al., 2013). The key role of WM in goal-directed behavior makes it a significant predictor of a number of skills and abilities ranging from fluid intelligence (Kane et al., 2005) to language learning (Baddeley et al., 1998), mathematical skills (Raghubar et al., 2010), and academic achievement (Gathercole and Pickering, 2000). Due to the critical role that WM plays in human behavior, considerable research effort has focused on describing its structure, that is, its cognitive building blocks and their interrelationships, in more detail. This has led to a plethora of models that share many features but also display important differences.

In broad terms, models of WM can be differentiated by their emphasis on content material (e.g., verbal and visuospatial) vs. constituent processes (e.g., updating and maintenance). With respect to content, previous behavioral, neuropsychological and neuroimaging research has consistently indicated that WM can be separated into verbal and visuospatial stores which mainly subserve maintenance functions (e.g., Smith and Jonides, 1999; Baddeley, 2002; Kane et al., 2004). However, consensus is lacking whether executive WM (e.g., attentional control, interference management, updating) is content-general or content-specific. Previous behavioral studies have produced mixed results: some studies support a more content-general view (Kane et al., 2004; Alloway et al., 2006), while others support content-specificity not only in maintenance but also in executive WM (e.g., Shah and Miyake, 1996; McKintosh and Bennett, 2003; Vuong and Martin, 2013). The content-general viewpoint has received some support from functional neuroimaging research (Chein et al., 2011), but a more recent comprehensive meta-analysis of neuroimaging data supports a model where executive WM is divided into dorsal “where” (visuospatial) and ventral “what” (verbal and object-based information) systems, thus indicating content-specificity (Nee et al., 2013). With respect to the processes that constitute WM, their number and quality have been discussed extensively. Suggested processes include, for example, combined storage, transformation, and coordination separate from supervision/mental speed (Oberauer et al., 2000); capacity, attention control, and secondary memory (Unsworth et al., 2014); inhibition, updating, and shifting (Miyake et al., 2000); and selection and updating (Bledowski et al., 2009). While many of the proposed process classifications listed above show overlap (e.g., attention control is closely related to inhibition), it is evident that a consensus is still lacking concerning the fundamental processes of WM.

Many of the studies listed above have employed factor analysis to investigate the functional structure of WM. The studies can be divided according to their respective analysis method into data-driven exploratory factor analyses (EFA) and hypothesis-driven confirmatory factor analyses (CFA), including structural equation modeling. In CFA, the fit of specific researcher-selected models is tested with the data. Given the existence of a number of theoretical models on WM, many relevant factor analytic studies have employed CFA to compare the explanatory value of several model architectures against their data. However, also EFA has been employed. The outcomes of previous factor analytical studies to determine the structure of WM have been mixed as to whether WM should primarily be divided according to the content material, the hypothetical processes, or both, or whether a single general latent WM factor accounts for much of the variance in WM behavior (e.g., Oberauer et al., 2000; Handley et al., 2002; Colom et al., 2006b; McCabe et al., 2010; Wilhelm et al., 2013; Dang et al., 2014). All in all, there is considerable variability in the outcomes of the previous factor analytical work on the structure of WM, and they fail to converge on whether WM should be described by content, by process, by a mixture of these factors, or as a single non-divisible system. There are several possible reasons for these discrepancies. For example, some researchers have limited their CFAs to certain model alternatives that did not cover all viable model options. Another key feature, which affects the results of any factor analysis, is the selection of tasks that are included in the analysis, and the test batteries in previous studies have varied considerably. Finally, somewhat limited sample sizes may also have affected some of the earlier factor solutions.

In the present study, we employed a latent factor approach using both CFA and EFA to investigate the structure of WM. EFA was included in order to control for possible confirmatory biases (i.e., not testing all viable models) because it allows for a model-free examination of candidate factors. In contrast to some earlier studies, we included an extensive WM test battery and a large and diverse adult sample. The present tasks represented typical hypothetical WM processes: simple span tasks have been argued to primarily tap WM maintenance (Kane et al., 2004), complex span tasks have been considered to reflect both maintenance and manipulation (Conway et al., 2005), and running memory tasks as well as *n*-back tasks are thought to measure higher-order WM processes, including updating and attention control (Morrison and Jones, 1990; Owen et al., 2005). With our CFAs, we sought to establish the functional separation between maintenance and updating division. With regard to content, each task paradigm was represented by two tasks variants: one consisting of numerical-verbal stimuli and one of visuospatial material. Thus, our test battery was designed to enable analyses of both content-based and a process-based latent structure.

## Materials and Methods

### Ethics Statement

The study was approved by the Joint Ethics Committee at the Departments of Psychology and Logopedics, Åbo Akademi University, and by the Human Research Review Board at the University of California, Riverside. Informed consent was obtained from all participants, participation was anonymous, and all participants were informed of their right to stop at any time.

### Participants

Participants were recruited through the online crowdsourcing forum Amazon Mechanical Turk (MTurk). MTurk has been shown to provide data with comparable quality to those obtained via traditional college student samples, while affording a more diverse and representative population (Berinsky et al., 2012; Casler et al., 2013; Goodman et al., 2013; Paolacci and Chandler, 2014). While we are not aware of similar research on WM that would have used MTurk workers, we aimed at recruiting active but not test-savvy participants by restricting our data collection to those who had completed more than 100, but less than 1000 work assignments (so-called HITs) (for possible effects of repeated testing, see Chandler et al., 2014). To minimize possible language-related issues, workers were restricted to the United States as identified by MTurk’s requirement of a United States bank location. To promote consistent and adequate data quality, a further restriction was that the participants were required to have a 95% work approval rating or higher (Peer et al., 2014).

Participants were paid $10 for the estimated 1.5–2 h participation in order to increase the recruitment rate of motivated participants and to provide compensation comparable to in-lab sessions. This rate of pay ($5–6.67 per hour) is well above the $1.38 median hourly wage that workers are willing to accept on MTurk (Horton and Chilton, 2010). At the end of the study, participants received a unique code to enter into the MTurk HIT to verify their participation. To ensure that each participant was a unique worker, a free online HTML scripting tool^1^ was used to track each participant HIT attempt and deny multiple attempts.

Altogether, 711 participants completed the entire study. 55 participants were excluded for having either missing values on the tasks (*n* = 4), for reporting the use of external aids such as note-taking during any of the WM tasks (*n* = 38), for spending over 1 day to complete the study (*n* = 1), and/or for being a multivariate outlier on task performance (*n* = 12) according to Mahalanobis distance [χ^2^ cutoff = 32.909, *df* = 12; note that the complex span task distractor tasks (see below) were included in this analysis]. Thus, the final sample consisted of 656 participants (see **Table 1** for demographic information)^2^.

**Table 1:** Demographic information of the study sample, *n* = 656.

Age in years	*M* = 33.50 (*SD* = 10.30), range = 18–71
Gender	58.8% female, 40.5% male, 0.6% other
Years of education	*M* = 15.69 (*SD* = 3.12), range = 0–30
Occupation status^a^	66.9% employed, 28.0% unemployed, 20.0% studying, 2.3% retired
QIDS total score	*M* = 6.61 (*SD* = 4.87), range = 0–26
QIDS score classification	48.9% none, 28.0% mild, 14.5% moderate, 5.0% severe, 1.1% very severe, 2.4% missing
Time spent on study (minutes)	*M* = 93.82 (*SD* = 33.52), range = 52–347

^a^*Participants could select multiple options regarding occupation status. QIDS, Quick Inventory of Depressive Symptomatology (Rush et al., 2003)*.

### Procedure

The study consisted of a background questionnaire and 10 WM tests. The entire study was administered online using an in-house developed web-based test platform that allows researchers to create, distribute, and manage psychological experiments. The platform employs a domain-specific programming language tailored to building psychological tasks, and it includes functions for handling the data, randomization, time measurement, and participant response. The experiment was conducted online by sending a link to the participants who completed the experiment on a computer of their choosing. All participants first completed the background questionnaire after which they completed the ten WM tests (average completion time: 1 h 34 min). The order of the WM tests was randomized for each participant in order to control for possible test order effects. The only exception to this rule was that the forward simple span task was always administered immediately before the backward simple span task. The participants were reminded several times not to use any external tools, such as note taking, during any of the tests, and they were queried about this at the end of the study (it was emphasized that their response would not have negative repercussions of any kind).

### Working Memory Tests

**FIGURE 1:**
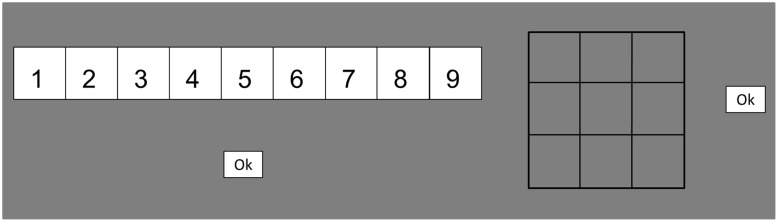
Response screens in the simple span tasks, complex span tasks, and running memory tasks. Numerical-verbal response screen on the left, visuospatial on the right. The “Ok” box was not present in the running memory tasks.

The WM test battery included ten WM tests that encompassed four different task paradigms, namely, simple span, complex span, running memory, and *n*-back tasks. All task paradigms were administered in two variants: one numerical-verbal variant involving the digits 1–9 and one visuospatial variant involving visuospatial locations within a 3 × 3 grid. Scores were calculated separately for the different tests and test variants. The numerical-verbal and visuospatial task variants were specifically designed to closely mirror each other in order to minimize the variance caused by stimulus-specific factors. The response screen was virtually identical in the respective verbal and visuospatial simple span, complex span, and running memory tasks, and consisted of the numbers 1–9 presented in a row of horizontally aligned boxes in the numerical-verbal tasks, and an empty 3 × 3 grid in their visuospatial equivalents (see **Figure 1**).

#### Simple Span Tasks

Simple span tasks are assumed to predominantly tap WM storage (Conway et al., 2005). In simple span tasks, lists of stimulus items with varying length are to be reproduced while maintaining the order of presentation. Both forward versions (repeating the list in the same order) and backward versions (repeating the list in the reverse order) have been used extensively in the literature, and they are part of common standardized neuropsychological and IQ tests (Wechsler, 1997a,b). Within the verbal domain, the backward version is generally more difficult than its forward counterpart, while the pattern is somewhat less clear when it comes to visuospatial material (Vandierendonck et al., 2004; Kessels et al., 2008; Monaco et al., 2013).

For the simple span tasks used here, stimulus lists (digits or spatial locations) of unpredictable length were presented. At the end of each list, participants were required to report the items in the exact order in which they had been presented in the forward version of the task, while the items were to be reported in the reverse order in the backward version of the task. Each test included two initial practice trials that consisted of one three-item list and one four-item list. In case of error, the practice trials were repeated until the participant answered correctly or until the practice was presented three times. This practice was followed by an additional practice trial consisting of a list with nine items (longest list length) to demonstrate the range. None of the practice trials were included in the dependent measures. The actual tests included seven trials involving list lengths ranging from three to nine.

All participants received the same set of lists; however, the lists were presented in a random order. The to-be-remembered item lists were pseudo-randomly generated in order to fulfill the following criteria: duplicate items (digits/locations) were not allowed to appear within the same list, directly ascending or descending items were not allowed to appear consecutively in the numerical-verbal version, while directly adjacent item triplets where not allowed to appear consecutively in the visuospatial version (e.g., the lower left matrix location followed by the lower middle matrix location followed by the central matrix location), ascending or descending odd or even item pairs were not allowed to appear consecutively in the numerical-verbal version, and only up to two identical items in the same serial position were allowed to appear in separate lists. Each item was presented for 1000 ms. In the verbal test, an asterisk was presented for 500 ms between every digit, while an empty matrix was presented for 500 ms between every item in the visuospatial test. At the end of each list, the response screen was displayed (see **Figure 1**). The participant selected the items by clicking on the digits or spatial locations displayed on the screen. No time limit was set to recalling the to-be-remembered items at the end of each list. The next list was presented once the participant clicked on an “Ok” box on the screen.

The total number of correctly recalled items, irrespective of list length and separately for the forward and backward tasks, was used as the dependent measure.

#### Complex Span Tasks

Complex span tasks were originally introduced to better capture WM capacity than the simple span tasks (Daneman and Carpenter, 1980) due to the demands they set on both storage *and* processing. In the complex span, the to-be-remembered span items are interleaved with processing requirements (e.g., mental arithmetic) that are not present in simple span tasks. Nowadays, complex span tasks have become one of the most commonly used measures of WM capacity in the research literature (Oberauer et al., 2012), especially since it has been argued that complex span tasks are better at predicting individual differences in higher cognitive functions than simple span (Engle et al., 1999). However, more recent studies have indicated that complex and simple span represent the same construct (Oberauer et al., 2000; Colom et al., 2006b), and that both predict Gf equally well, especially when certain issues in, for example, administration (implementing no discontinue rule) and scoring (including variability from all lists) are taken into account (Colom et al., 2006a; Unsworth and Engle, 2007). Nevertheless, given that both task types are actively used in the current literature, both simple and complex span tasks were included in the present study.

**FIGURE 2:**
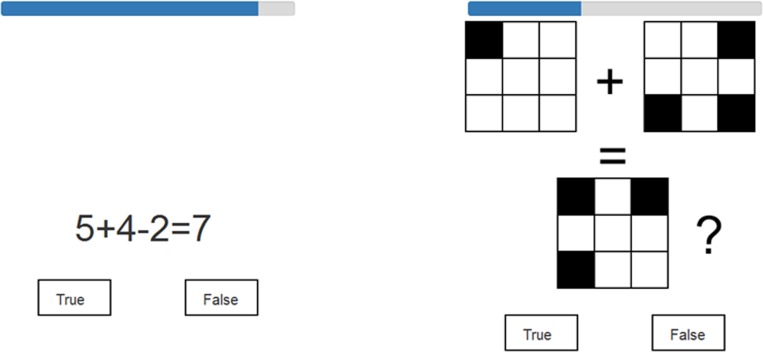
Examples of distractor items in the complex span tasks. Numerical-verbal example item on the left, visuospatial on the right. A timer bar above each item depicts the remaining response time.

For the complex span tasks, stimulus lists of unpredictable length were presented. As in the forward simple span, the participant was required to recall the items in the same order as they were presented. However, after each to-be-remembered item, the participant had to make a true/false judgment about a distractor item (for examples, see **Figure 2**). At the end of each list, the participant was required to report the to-be-remembered stimuli in the exact order in which they had been presented. In the numerical-verbal version, the distractor items consisted of simple arithmetic problems involving additions and subtractions. Each arithmetic item required the performance of two operations on a single-digit number that was between two and nine. The suggested responses varied between one and eleven. Incorrect suggestions were numbers within 1–3 in numerical value of the true answer. In the visuospatial version, the distractor items required participants to mentally combine two 3 × 3 matrix patterns in order to decide whether their combination corresponded to a suggested third pattern. Each to-be-combined matrix pattern included 1–4 filled matrix locations, and the final actual combination included 3–6 filled locations. Incorrect suggestions deviated from correct answers in total squares by at most one, but the deviation could also be due to only the spatial placement of filled locations (in which case one square was incorrectly placed). A blue timer bar displayed the remaining time to solve each distractor item (see **Figure 2**). The task continued automatically to the next to-be-remembered item once a true or false button was clicked or once a maximum of 6 s had elapsed on a distractor item. The to-be-remembered items and distractor items were always from the same stimulus category (i.e., digits or matrices) in order to maximize the likelihood of participants employing only content-specific processing during each respective complex span task. Each test included two initial practice trials that consisted of one list with three items and one list with four items. The practice trials were repeated until the participant gave the correct answers or until the practice trials were presented three times. The practice trials were followed by an additional practice trial with a list length of seven (longest list length) to familiarize participants with the range of list lengths. None of the practice trials were included in the dependent measures. The actual tests included five trials that consisted of list lengths ranging from three to seven.

All participants received the same set of lists; however, the lists were presented in a random order. The to-be-remembered item lists were pseudo-randomly generated in an identical fashion as for the simple span tasks. The task progressed as follows: fixation point (500 ms), to-be-remembered item (1000 ms), fixation point (500 ms), distractor item (up to 6000 ms), and this sequence was looped until the end of a list. At the end of each list, the response screen was displayed (see **Figure 1**). Participants selected items by clicking on the corresponding digits or spatial locations. No time limit was set to recalling the to-be-remembered items at the end of each list. The next list was presented once the participant clicked on an “Ok” box on the screen.

The total number of correctly recalled items, irrespective of list length, was used as the dependent measure.

#### Running Memory Tasks

The running memory task was first introduced by Pollack et al. (1959). In the running memory task, a list of items is presented, and the participant is required to recall the n last items in correct order once the list ends. As the list length is unknown to the participant, the last *n* items should be constantly updated, making the running memory task a prototypical measure of WM updating. Nonetheless, there is some controversy around whether or not running memory performance actually requires active updating (see e.g., Elosúa and Ruiz, 2008; Broadway and Engle, 2010; Botto et al., 2014). However, regardless of whether participants perform the running memory task by using an active or passive strategy, Broadway and Engle (2010) observed that running memory correlated well with both complex span tasks and Gf.

For the running memory tasks used here, stimulus lists of unpredictable length were shown. At the end of each list, the participant was required to report the last four items in the exact order in which they had been presented. Each test included two practice trials that consisted of one five-item list and one six-item list. The actual test started once the participant answered correctly on both of the practice trials or once a total of three attempts at the practice trials had been made. The actual test included eight lists that consisted of 4–11 items (one trial per list length).

All participants received the same set of lists; however, the lists were presented in a random order. The item lists were pseudo-randomly generated to fulfill the following criteria: the same item (digit/location) was only allowed to appear twice in a given list, the same item was not allowed to appear consecutively, directly ascending or descending items were not allowed to appear consecutively in the numerical-verbal version, while directly adjacent item triplets where not allowed to appear consecutively in the visuospatial version, and only up to two identical items in the same locations were allowed to appear within the target items in separate lists. Each item was presented for 1000 ms. In the verbal test, an asterisk was presented for 500 ms between every item, while the matrix was empty for 500 ms in the visuospatial test. At the end of each list, the response screen was displayed (see **Figure 1**). However, here the “Ok” box was not present as the program required a full four-item response in order to proceed to the next list. In the spatial test, the text “Please respond” was inserted on the screen in order to clearly indicate that a response was required. The participant selected items by clicking on the digits or spatial locations presented on the screen. Participants had no time limit while recalling the to-be-remembered items at the end of each list.

The number of correctly recalled items was used as the dependent measure; however, the list with only four items was excluded as it does not require any updating.

#### *N*-Back Tasks

In this task, participants are required to indicate whether the currently presented item matches an item that was presented *n* steps back. Thus, WM updating is assumed to be critical for successful performance on this task. The *n*-back task has been especially popular in neuroimaging research, but the task has been noted to correlate only weakly with other WM tasks, especially complex span tasks, raising questions as to what the *n*-back measures (e.g., Kane et al., 2007; Miller et al., 2009; Jaeggi et al., 2010a; Redick and Lindsey, 2013). Latent variable studies have, however, indicated that the *n*-back is more closely related to the complex span than previously suggested (see Schmiedek et al., 2014, and also Schmiedek et al., 2009; Wilhelm et al., 2013).

The *n*-back tests used here consisted of a 1- and 2-back task. In the 1-back task, the participant was to respond whether the currently visible item was the same (target), or not (no-target), as the previous item by pressing the N (target) and M (no-target) keys on the computer keyboard. In the 2-back task, the participant was required to indicate whether the currently presented item was the same as the item that was presented two steps back. The order of the actual tasks (1-back or 2-back) was randomized for every participant. Both tasks were preceded by a corresponding practice block that consisted of twelve items [four targets, four no-targets, and four lures (see below)]. Each practice block was administered up to three times, or until two out of four target items and half of the total items were answered correctly.

All participants received the same set of items. The item lists for the actual tests were pseudorandomly generated in order to include 16 target items, 16 no-target items, and 16 so-called lure items (i.e., 48 responses per task). Lure items in the 1-back task were n+1 items, that is, items that matched the item that was presented two steps back (e.g., in the list 4-8-3-8, the last 8 is an n+1 lure). In the 2-back task, lure items consisted of n+1 (*n* = 4), n-1 (*n* = 4), and n+ and -1 (*n* = 8, e.g., in the list 4-2-4-4, the last 4 is an n+ and -1 lure) items. Of the target items, three items also matched the item presented three steps back, three items also matched the item presented one step back, and ten items only matched the target item (when considering the most recently presented items). Each item was presented for 1500 ms. In the verbal test, an asterisk was presented for 450 ms between every digit, while the matrix was empty for 450 ms between every item in the visuospatial test. The participant had 1950 ms (item presentation + fixation) to respond to each item.

The proportion of hits (correct targets) minus the proportion of false alarms (“same” responses on no-target items) on the 2-back task was used as the dependent measure. We did not use the 1-back tasks as outcome measures due to accuracy rates being close to ceiling which distorted distributions (numerical-verbal *M* = 90.00%, skewness = -3.03, kurtosis = 9.86; visuospatial *M* = 87.21%, skewness = -2.32, kurtosis = 5.64).

### Statistical Analyses

Factor analysis was used to investigate the latent structure of the data. Prior to performing the factor analyses, the dependent variables were Box-Cox transformed in order to improve normality by decreasing the skewness of the distributions (Osborne, 2010, see **Table 2**). The CFAs were performed with MPlus version 7.4. The CFA models were estimated using maximum likelihood with the Satorra-Bentler rescaled chi-square statistic (Satorra and Bentler, 1994) due to multivariate non-normality (Korkmaz et al., 2014). The models were parameterized by fixing factor means to 0 and variances to 1.

**Table 2:** Descriptive statistics and reliability estimates for each of the WM accuracy rate measures.

Variable	*M* (*SD*)	Skewness BT	Kurtosis BT	Skewness AT	Kurtosis AT	α
**Numerical-verbal**						
SSTF	73.19 (17.06)	-0.79	1.23	0.01	-0.56	0.66
SSTB	62.93 (18.66)	-0.18	-0.17	0.00	-0.42	0.71
CST	70.96 (29.01)	-0.99	-0.10	0.00	-1.43	0.83
RMT	70.49 (21.32)	-0.94	0.79	-0.01	-0.78	0.72
2-back	57.68 (26.37)	-0.82	0.43	-0.00	-0.82	0.98

**Visuospatial**						
SSTF	62.06 (18.37)	-0.36	0.20	0.05	-0.27	0.71
SSTB	64.66 (20.13)	-0.91	0.82	-0.02	-0.46	0.79
CST	42.15 (30.34)	0.37	-0.96	0.03	-1.02	0.85
RMT	54.15 (24.35)	-0.33	-0.67	0.02	-0.77	0.77
2-back	53.04 (30.23)	-0.66	-0.04	0.01	-0.94	0.98

*SSTF, simple span task forward; SSTB, simple span task backward; CST, complex span task; RMT, running memory task; BT, before Box-Cox transformation; AT, after Box-Cox transformation; α, Cronbach’s alpha. Test scores are provided in percent correct, except for the 2-back tasks, where we employed the corrected recognition score, i.e., percent hits minus percent false alarms. *n* = 656*.

Several fit indices were used to assess model fit. The χ^2^-statistic shows the magnitude of discrepancy between the model-implied and the observed data matrix where a non-significant result (*p* > 0.05) indicates a well-fitting model. However, the power of the χ^2^-statistic is directly related to sample size, and thus a trivial discrepancy may lead to the rejection of a model in large samples. Therefore, we also report and interpret additional fit indices. The Root Mean Square Error of Approximation (RMSEA) is an absolute measure of fit following a non-central χ^2^-distribution, which allows for discrepancies between estimated and observed covariances as a function of degrees of freedom. RMSEA favors parsimonious models (more degrees of freedom) and large sample sizes (Kline, 2011). The Comparative Fit Index (CFI) builds on the relative difference between the non-centrality parameters (i.e., the χ^2^ statistic minus degrees of freedom) of the estimated model and a baseline independence model (Bentler, 1990). The Standardized Root Mean Square Residual (SRMR) is a measure of the mean absolute correlation residual, that is, the overall difference between estimated and observed standardized covariances (Kline, 2011). Cut-off levels for approximate fit indexes considered to indicate an acceptable model fit were RMSEA < 0.08, CFI ≥ 0.90, and SRMR = < 0.05 (Hooper et al., 2008). Akaike (AIC) and Bayesian (BIC) information criteria allows for comparisons of non-nested models, where the model with the lowest value is preferred. Both criteria are based on minus two times the loglikelihood value, and favor parsimony by adding a penalty term of the number of estimated parameters multiplied by 2 (AIC) or by the natural logarithm of N (BIC).

An EFA was also used to investigate the latent factor structure of the data from a data-driven perspective. The EFAs were conducted with IBM SPSS version 21.0.0.0 using principal axis factoring with oblique Promax rotation (Osborne and Costello, 2009).

## Results

Descriptive statistics for the WM tasks are summarized in **Table 2**, and task intercorrelations are presented in **Table 3**. It is important to note that the task reliabilities range between acceptable and very high values, despite the fact that the tasks were fairly short and that participants completed the tasks at home without experimenter explanation or supervision. The reliabilities are also comparable to those of previous laboratory-based studies (e.g., Engle et al., 1999).

**Table 3:** Test intercorrelations (Pearson two-tailed).

		Numerical-verbal					Visuospatial				
		SSTF	SSTB	CST	RMT	2-back	SSTF	SSTB	CST	RMT	2-back
**Numerical-verbal**	SSTF	–									
	SSTB	0.55	–								
	CST	0.41	0.44	–							
	RMT	0.35	0.45	0.31	–						
	2-back	0.21	0.29	0.31	0.26	–					

**Visuospatial**	SSTF	0.32	0.32	0.28	0.28	0.37	–				
	SSTB	0.27	0.36	0.35	0.29	0.44	0.56	–			
	CST	0.30	0.39	0.34	0.31	0.37	0.46	0.40	–		
	RMT	0.22	0.33	0.30	0.33	0.39	0.42	0.44	0.43	–	
	2-back	0.23	0.29	0.36	0.27	0.57	0.33	0.39	0.39	0.40	–

*SSTF, simple span task forward; SSTB, simple span task backward; CST, complex span task; RMT, running memory task. The correlations were calculated using the Box-Cox transformed test data. *n* = 656 in all correlations. All correlations are significant at *p* < 0.01*.

### Confirmatory Factor Analyses

**FIGURE 3:**
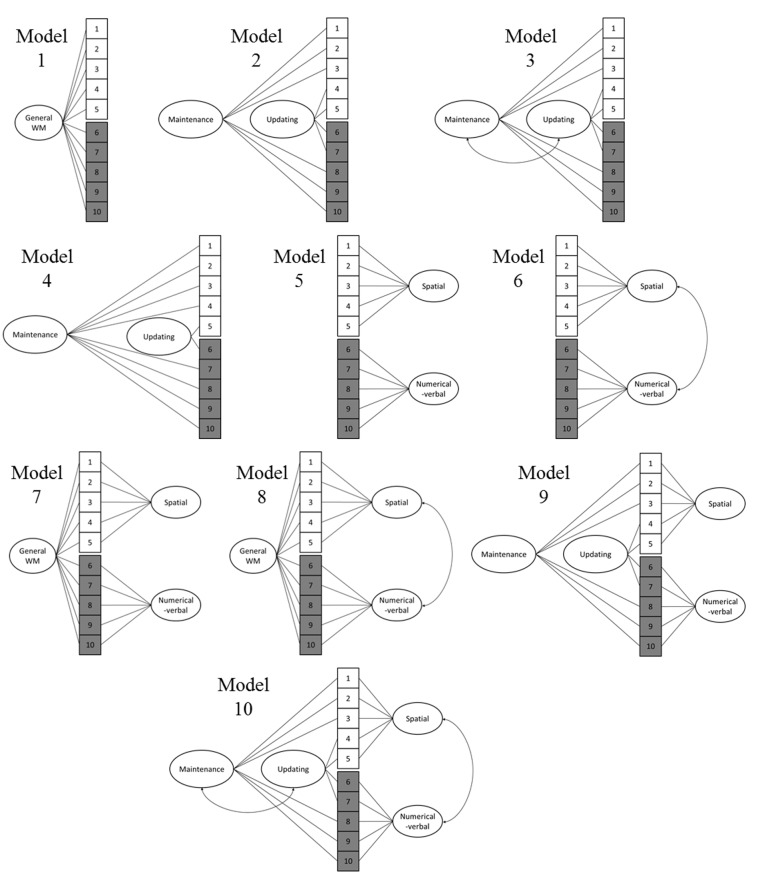
Models tested with confirmatory factor analysis. 1 = Visuospatial simple span forward, 2 = Visuospatial simple span backward, 3 = Visuospatial complex span task, 4 = Visuospatial running memory task, 5 = Visuospatial 2-back, 6 = Numerical-verbal 2-back, 7 = Numerical-verbal running memory task, 8 = Numerical-verbal complex span task, 9 = Numerical-verbal simple span backward, 10 = Numerical-verbal simple span forward.

On the basis of previous empirical and theoretical work, we tested 10 different models that are graphically depicted in **Figure 3**. We focused on two divisions, one content-based and one process-based, both of which are prominent in previous research: visuospatial vs. numerical-verbal, as well as maintenance vs. updating. Nevertheless, Model 1 consisted of a single general WM factor that encompassed all the WM tests, following the alternative view of WM as a unitary capacity. Model 2 included two latent process factors: maintenance (all simple and complex span tasks) vs. updating (all running memory and 2-back tasks). Model 3 was identical to Model 2, except that the two process factors were correlated to represent facilitation between the two processes (e.g., effective updating of new items should support the maintenance and rehearsal of the items in question). Model 4 involved a process distinction where only the *n*-back tasks represent updating (for the discussion concerning the role of active updating in running memory tasks, see e.g., Broadway and Engle, 2010), while all other tasks represent maintenance. Model 5 included two content factors: one visuospatial and one numerical-verbal. Model 6 was identical to Model 5, except that the latent visuospatial and numerical-verbal factors were correlated to represent facilitation across content domains, for example, through verbalization of visuospatial items. Model 7 represented a facet model that included the abovementioned content factors and one general factor that loaded on all tasks. Again, Model 8 was identical to Model 7, except that the visuospatial and numerical-verbal factors were correlated. Model 9 also represented a facet model that included the abovementioned content factors as well as the two process factors (for this specific model, the number of iterations was increased from 1000 to 5000 in order to achieve convergence). Model 10 was identical to Model 9, except that the two content factors were correlated and the process factors were correlated (for detailed model scripts and outputs, see Supplementary Material). We originally planned to also test a model that included uncorrelated content factors (visuospatial and numerical-verbal) with a hierarchically superordinate factor reflecting a central executive (and also a nearly identical model where the content factors were correlated), but this model could not be identified (i.e., did not converge) with the present data set due to the limited number of indicators of the hierarchically superordinate general WM factor.

**FIGURE 4:**
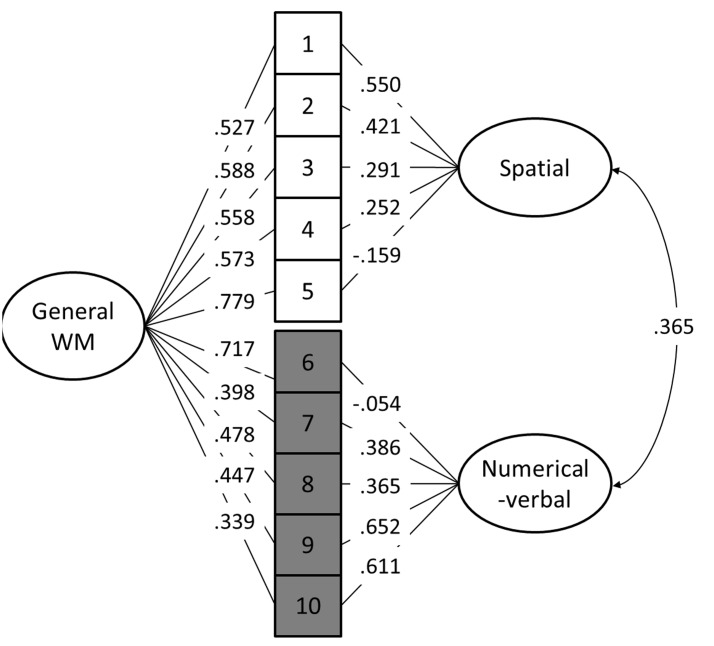
Best fitting structural equation model (Model 8). 1 = Visuospatial simple span forward, 2 = Visuospatial simple span backward, 3 = Visuospatial complex span task, 4 = Visuospatial running memory task, 5 = Visuospatial 2-back, 6 = Numerical-verbal 2-back, 7 = Numerical-verbal running memory task, 8 = Numerical-verbal complex span task, 9 = Numerical-verbal simple span backward, 10 = Numerical-verbal simple span forward.

Out of the 10 models, Models 8 and 10 provided the best fit to the data (**Table 4**). When comparing model 10 to model 8 by constraining the correlation between the maintenance and updating factors to 1, model 8 did not fit the data significantly worse [Satorra-Bentler scaled χ^2^(1) = 0.05, *p* = 0.82]. Additionally, Model 8 had the lowest AIC and BIC values. Therefore, Model 8 was interpreted to be a more parsimonious indicator of the latent structure (see **Figure 4**). It is noteworthy that in this model, the 2-back tasks did not load significantly on their respective content factors.

**Table 4:** Model fit indexes of the 10 tested models with best fitting model boldfaced.

		Chi-square test of model fit							RMSEA				
Model	Number of estimated parameters	Value	*df*	*p*	Scaling correction factor for MLR	CFI	TLI	SRMR	Est.	90% CI	Pr. RMSEA ≤ 0.05	ΔAIC	ΔBIC
1	30	331.495	35	0.0000	1.0523	0.840	0.794	0.062	0.114	0.103–0.125	0.000	275.4	226.1
2	30	646.463	35	0.0000	1.0423	0.669	0.575	0.214	0.163	0.152–0.174	0.000	600.4	551.1
3	31	308.529	34	0.0000	1.0446	0.852	0.804	0.061	0.111	0.100–0.122	0.000	250.9	206.1
4	31	250.776	34	0.0000	1.0538	0.883	0.845	0.055	0.099	0.087–0.110	0.000	192.9	148.1
5	30	550.693	35	0.0000	1.0446	0.721	0.642	0.211	0.150	0.139–0.161	0.000	501.9	452.6
6	31	278.573	34	0.0000	1.0277	0.868	0.825	0.063	0.105	0.094–0.116	0.000	214.9	170.1
7	40	69.546	25	0.0000	1.0229	0.976	0.957	0.030	0.052	0.038–0.067	0.382	17.8	13.3
**8**	**41**	**50.914**	**24**	**0.0011**	**1.0088**	**0.985**	**0.973**	**0.022**	**0.041**	**0.025–0.057**	**0.804**	**0**	**0**
9	40	402.494	25	0.0000	0.9761	0.796	0.633	0.177	0.152	0.139–0.165	0.000	339.5	335.0
10	42	50.906	23	0.0007	1.0079	0.985	0.970	0.022	0.043	0.027–0.059	0.747	1.9	6.4

*Df, degrees of freedom; CFI, Comparative Fit Index; TLI, Tucker-Lewis Index; SRMR, Standardized Root Mean Square Residual; RMSEA, Root Mean Square Error of Approximation; Est., estimated value of RMSEA; AIC, akaike information criterion; BIC, Bayesian information criterion, ΔAIC and ΔBIC represent the difference between the model’s values and the best fitting model’s values*.

### Exploratory Factor Analyses

To complement the CFAs with a data-driven approach, we explored the factor structure of the complete dataset (including all 10 WM measures) using EFA. The Kaiser-Meyer-Olkin measure of sampling adequacy was 0.87, Bartlett’s test of sphericity was significant, χ^2^(45, *N* = 656) = 2027.39, *p* < 0.001, and the diagonal values of the anti-image correlation matrix were all above 0.8, suggesting that the data were adequate for factor analysis. A Scree test (see e.g., Zwick and Velicer, 1986) indicated the extraction of two or three factors. We report both solutions due to their perceived relevance, even though the two-factor solution was supported by parallel analysis (Horn, 1965; Hayton et al., 2004) and the Kaiser criterion (i.e., Eigenvalues > 1). The resulting factor loadings in the two- and three-factor pattern matrices are presented in **Table 5**, along with the correlations between the resulting factors. The two-factor model accounted for 54.25% of the variance while the three-factor model accounted for an additional 8.38%. In the two-factor model, the first factor was interpreted to jointly reflect visuospatial WM and *n*-back, while the second factor encompassed numerical-verbal WM. The three-factor model was similar to the two-factor model, but here the *n*-back tasks represented their own factor.

**Table 5:** Exploratory factor analysis with all 10 WM measures: factor loadings (loadings > 0.30 are boldfaced), commonalities, and factor correlations.

	Two-factor solution			Three-factor solution			
	Factor 1	Factor 2	Communality	Factor 1	Factor 2	Factor 3	Communality
Visuospatial SSTF	**0.58**	0.09	0.41	-0.04	**0.90**	-0.13	0.63
Visuospatial SSTB	**0.65**	0.05	0.47	0.01	**0.63**	0.11	0.51
Visuospatial CST	**0.51**	0.17	0.40	0.18	**0.37**	0.17	0.39
Visuospatial RMT	**0.61**	0.04	0.41	0.06	**0.39**	0.25	0.39
Visuospatial 2back	**0.69**	-0.05	0.43	-0.01	-0.09	**0.86**	0.63
Numerical-verbal 2back	**0.74**	-0.10	0.46	-0.05	0.11	**0.66**	0.51
Numerical-verbal RMT	0.15	**0.44**	0.30	**0.46**	0.07	0.07	0.30
Numerical-verbal CST	0.21	**0.44**	0.35	**0.48**	-0.02	0.21	0.37
Numerical-verbal SSTB	-0.02	**0.81**	0.63	**0.84**	-0.02	-0.04	0.64
Numerical-verbal SSTF	-0.11	**0.76**	0.49	**0.74**	0.01	-0.12	0.48
Factor 1	1			1			
Factor 2	0.63	1		0.62	1		
Factor 3	NA	NA		0.55	0.68	1	

*SSTF, simple span task forward; SSTB, simple span task backward; CST, complex span task; RMT, running memory task. *n* = 656*.

## Discussion

We set out to explore the latent structure of WM by administering an extensive test battery to a large sample of adult participants. Our focus was on two fundamental distinctions of WM, namely whether the structure of WM is driven by content-specific and/or process-based factors. The analyses provided several interesting results. Our most robust finding, observed in both the best fitting CFA model and the EFAs, is that the content-based division (spatial, numerical-verbal) is a pervasive aspect of the WM system. Starting from the multicomponent model by Baddeley and Hitch (1974), our data are in line with a number of studies and theoretical accounts that divide WM according to this particular content division. However, the associations between our tasks and the two content factors were not uniform as the *n*-back tasks did not load on their respective content factors in either our best fitting CFA or in the three-factor EFA solution. One reason for this discrepancy might be related to the retrieval demands in the various tasks: the simple span, running memory, and complex span tasks all require free recall at the end of each list, whereas the *n*-back task requires speeded recognition (Kane et al., 2007). Of future interest would be to investigate the association between the *n*-back task and other speeded and/or recognition-based WM tasks (see e.g., Parmenter et al., 2006), especially considering that the *n*-back has been criticized for a lack of convergent validity with the complex span (Redick and Lindsey, 2013; however, see Schmiedek et al., 2009, 2014).

Although the *n*-back tasks did not load on the content factors in the CFA, they did load on the general WM factor. This, together with the EFA factor intercorrelations, indicates that the *n*-back shares a significant amount of variance with the other WM tasks. We can, however, only speculate what this general factor represents. First, it may represent a general aspect of the WM system such as the central executive (Baddeley and Hitch, 1974) or the focus of attention (Cowan, 1999). The fact that the *n*-back tasks load highly with this factor might be related to the nature of the *n*-back tasks which, in contrast to the other WM paradigms employed here, require continuous monitoring and decision-making. Second, the general WM factor could encompass fluid intelligence that is related to WM (Conway et al., 2002; Jaeggi et al., 2010b; Shelton et al., 2010). According to this hypothesis, the highest loadings of the *n*-back tasks on this factor might be related to their higher inherent novelty, as participants probably had less experience with this type of task than with active recall tasks. Also, visuospatial tasks in general tend to have a higher novelty value than verbal ones (perhaps even affecting their proneness to compensatory strategies such as chunking), which is reflected by the current loadings on the general WM factor (Baddeley, 1996; Miyake et al., 2001). Third, the general WM factor could be a combination of some unique *n*-back features coupled with elements of content-general executive attention and/or fluid intelligence. This interpretation would conform to the current three-factor EFA model where the *n*-back factor has a higher correlation with the visuospatial than the numerical-verbal factor (*r* = 0.68 and 0.55, respectively). Previous research has indicated that even verbal *n*-back tasks recruit spatial processes (Meegan et al., 2004) which this interpretation would seem to support. Furthermore, some *n*-back training work has shown that the most consistent transfer effects are observed in visuospatial domains, regardless of whether the *n*-back training consists of spatial and/or verbal material (Colom et al., 2013; Jaeggi et al., 2014; Au et al., 2015, 2016, but see Soveri et al., 2017). Also, Redick and Lindsey (2013) noted in their meta-analysis that the correlation between *n*-back and complex span is greater when the complex span is non-verbal and lowest when both rely on verbal stimuli, which may also indicate spatial processing in the *n*-back irrespective of stimulus materials. The nature of a possible spatial component in verbal *n*-back is not clear, but it might be related to the use of spatial strategies (encoding the stimulus sequence as an unfolding row of items in space) to keep track of the item positions.

In contrast to the robust division into visuospatial and numerical-verbal WM, we failed to find support for a distinction between maintenance (represented by simple and complex span tasks) and updating (represented by running memory and *n*-back) in either our CFAs or EFAs, albeit this process-based distinction has been prominent in WM research. It could be that such a distinction is indeed non-existent (cf. Schmiedek et al., 2009), or the two processes are too closely related to be differentiated in the current setup, or the present task selection was not optimal for the emergence of such a distinction. As to the last alternative, the current tasks might have been more similar in their processing demands in comparison to the single verbal-visuospatial content distinction that divided the battery of tasks in two equal halves. The issue of task selection concerns every factor analytical study, as the extracted factor structure is dependent on the measures that are fed into the analysis. Future work is needed to replicate our results with different task constellations and paradigms. Systematic replication attempts of previous models are not that common, although it is highly crucial in order to ascertain the generality, rather than sample- or task-specificity, of a model.

On a different note, the present study demonstrates the feasibility of online data collection in obtaining larger and demographically more diverse participant samples. Our findings revealed robust effects of WM load (1-back vs. 2-back, and digit span forward vs. backward)^3^, and most importantly, they showed comparable task reliabilities as has been observed in the laboratory (e.g., Engle et al., 1999; Conway et al., 2002). Furthermore, earlier online cognitive studies have provided results that are comparable to laboratory findings (Germine et al., 2012; Crump et al., 2013; Enochson and Culbertson, 2015). However, possible error variance resulting from the uncontrolled testing conditions cannot be dismissed.

A limitation concerning the current study should be mentioned. The numerical-verbal complex span task was strongly negatively skewed, and 126 out of the 656 participants obtained a maximum score. One might suspect that this reflects cheating on the task; however, such a pattern was not observed in any of the other span tasks, which would seem to contradict this suspicion. Instead it might be that the interfering items were too easy and/or too much time was allotted to solving each interfering item (6 s per interfering task), which possibly enabled rehearsal of the to-be-remembered span items.

## Conclusion

The present results indicate that a fundamental division in WM goes along its contents. With our test battery, this emerged as numerical-verbal and visuospatial factors, but it is also possible that it is better characterized as a “what” and “where” distinction where the former encompasses both verbal and object information, and the latter encompasses spatial information (Nee et al., 2013). Our results also indicate that all the measured WM tasks share a significant amount of variance, which suggests the presence of a general WM factor that possibly reflects content-general attention or fluid intelligence needed for performing novel tasks. Finally, the *n*-back tasks exhibited some unique features: they loaded more strongly on the visuospatial domain (irrespective of stimulus materials), and especially on a general WM factor in the final CFA model. We speculated that this pattern of *n*-back results may relate to the use of visuospatial strategies in solving all *n*-back tasks, higher demands on executive/attentional resources, or higher task novelty that calls for fluid intelligence in finding optimal ways to perform the task. Given the present findings and previous studies, it appears that a content-based numerical-verbal vs. visuospatial division of WM is more robust than process-based divisions such as maintenance vs. updating.

## Author Contributions

Conceived and designed the research: OW, AS, MA, RCH, SMJ, ARS, ML. Aggregated the data: OW, RCH. Analyzed the data: OW, DV. Wrote the original draft: OW, RCH, DV. Provided critical revisions: OW, AS, DV, SMJ, ARS, ML. All authors approved the final version of the manuscript for submission.

## Conflict of Interest Statement

SMJ has an indirect financial interest in MIND Research Institute, whose interest is related to this work. The other authors declare that the research was conducted in the absence of any commercial or financial relationships that could be construed as a potential conflict of interest.

## Footnotes

1 http://uniqueturker.myleott.com/

2 A more restricted sample consisting of 360 participants that remained after an extensive exclusion due to several background factors (e.g., medical diagnoses and depression scores), univariate outlier analyses using Leys’ method (Leys et al., 2013), and the current exclusion criteria, yielded very similar EFA results, and therefore, only the larger sample was used here.

3 Paired samples *t*-tests Verbal Simple span task Forward vs. Backward, *t*(394) = 12.38, *p* < 0.001; Verbal 1-back vs. 2-back, *t*(394) = 19.83, *p* < 0.001; Visuospatial 1-back vs. 2-back, *t*(394) = 16.38, *p* < 0.001.
